# Efficacy and safety of pyronaridine–artesunate versus artemether–lumefantrine in the treatment of acute uncomplicated malaria in children in South-West Nigeria: an open-labelled randomized controlled trial

**DOI:** 10.1186/s12936-023-04574-7

**Published:** 2023-05-13

**Authors:** Catherine O. Falade, Adebola E. Orimadegun, Fiyinfoluwa I. Olusola, Obaro S. Michael, Oluwafunmibi E. Anjorin, Roland I. Funwei, Aduragbenro D. Adedapo, Abiola L. Olusanya, Bose E. Orimadegun, Olugbenga A. Mokuolu

**Affiliations:** 1 Department of Pharmacology and Therapeutics, College of Medicine, University of Ibadan, Ibadan, Nigeria; 2 Institute for Advanced Medical Research and Training, College of Medicine, University of Ibadan, Ibadan, Nigeria; 3 Institute of Child Health, College of Medicine, University of Ibadan, Ibadan, Nigeria; 4 Department of Accident and Emergency, Obafemi Awolowo University Teaching, Hospital, Ile-Ife, Nigeria; 5 Department of Pharmacology, Babcock University, Ilisan, Remo, Ogun State Nigeria; 6 Department of Chemical Pathology, College of Medicine, University of Ibadan, Ibadan, Nigeria; 7 Department of Paediatrics, University of Ilorin Teaching Hospital, Ilorin, Nigeria

**Keywords:** Randomized clinical trial, Pyronaridine–artesunate, Artemether–lumefantrine, Efficacy and safety, Uncomplicated malaria, Nigeria

## Abstract

**Background:**

In Nigeria, declining responsiveness to artemether–lumefantrine (AL), the artemisinin-based combination therapy (ACT) of choice since 2005, has been reported. Pyronaridine–artesunate (PA) is a newer fixed-dose ACT recently prequalified by the WHO for the treatment of uncomplicated falciparum malaria. However, PA data from the Nigerian pediatric population is scarce. Therefore, the efficacy and safety of PA and AL using the WHO 28-day anti-malarial therapeutic efficacy study protocol in Ibadan, southwest Nigeria, were compared.

**Methods:**

In an open-labelled, randomized, controlled clinical trial, 172 children aged 3–144 months with a history of fever and microscopically confirmed uncomplicated *Plasmodium falciparum* malaria were enrolled in southwest Nigeria. Enrollees were randomly assigned to receive PA or AL at standard dosages according to body weight for 3 days. Venous blood was obtained for hematology, blood chemistry, and liver function tests on days 0, 3, 7, and 28 as part of the safety evaluation.

**Results:**

165 (95.9%) of the enrolled individuals completed the study. About half (52.3%; 90/172) of enrollees were male. Eighty-seven (50.6%) received AL, while 85 (49.4%) received PA. Day 28, adequate clinical and parasitological response for PA was 92.7% [(76/82) 95% CI 83.1, 95.9] and 71.1% [(59/83) 95% CI 60.4, 79.9] for AL (0.001). Fever and parasite clearance were similar in both groups. Two of six and eight of 24 parasite recurrences were observed among PA- and AL-treated children, respectively. PCR-corrected Day-28 cure rates for PA were 97.4% (76/78) and 88.1% (59/67) for AL (= 0.04) in the per-protocol population after new infections were censored. Hematological recovery at day 28 was significantly better among PA-treated patients (34.9% 2.8) compared to those treated with AL (33.1% 3.0) (0.002). Adverse events in both treatment arms were mild and similar to the symptoms of malaria infection. Blood chemistry and liver function tests were mostly within normal limits, with an occasional marginal rise.

**Conclusion:**

PA and AL were well-tolerated. PA was significantly more efficacious than AL in both the PCR-uncorrected and PCR-corrected per-protocol populations during this study. The results of this study support the inclusion of PA in the anti-malarial treatment guidelines in Nigeria.

**Retrospective trial registration:**

Clinicaltrials.gov: NCT05192265.

**Supplementary Information:**

The online version contains supplementary material available at 10.1186/s12936-023-04574-7.

## Background

Despite efforts to control and eliminate malaria, it remains a major public health concern in sub-Saharan Africa (SSA). As documented in successive editions of the World Malaria Report, malaria continues to cause unacceptably high levels of disease and death in sub-Saharan Africa. According to the 2021 World Health Organization (WHO) malaria report, there were an estimated 241 million malaria cases and 627,000 malaria deaths in 2020, representing about 14 million more cases in 2020 compared to 2019 and 69,000 more deaths [1]. Artemisinin-based combination therapy (ACT), the current WHO global standard for the treatment of acute uncomplicated malaria [1], has contributed to a significant reduction in malaria prevalence in recent years [2]. This progress is endangered, however, by the emergence of *Plasmodium falciparum* strains with reduced in vivo susceptibility to artemisinin, which was first reported in Western Cambodia [3, 4], as well as reduced political commitment and funding for malaria control [2].

Since the first report of reduced in vivo susceptibility in the late 2000s, the prevalence of drug-resistant infections has increased and spread to other parts of Southeast Asia and the rest of the world [2, 3, 5]. Artemisinin resistance, which is strongly linked to a mutation in the propeller region of the *P. falciparum* Kelch protein gene on chromosome 13 (Kelch13), was first discovered in western Cambodia and has since spread to most malaria-endemic regions around the world, including Africa and India [6–9]. Previous research found a lack of selection for Kelch13 in southwest Nigeria [10]. Another study also recorded low polymorphism in samples from Kano, Nigeria, indicating a lack of selection for the *kelch 13* gene [11].

Artemether–lumefantrine (AL), the most used treatment for uncomplicated malaria in several African countries, including Nigeria, has an impressive safety and efficacy record [12, 13]. However, it must be taken twice daily and consumed with a fatty meal for optimal absorption, and the relatively short half-life puts patients at risk of reinfection [14]. There is a need to expand options for anti-malarial treatment, first to delay resistance, contribute to resistance management where this is already a problem, and reduce drug pressure on AL. Pyronaridine–artesunate (PA) does not have the limitations of AL and could reduce the pressure on AL, thus making it a viable option to evaluate.

Pyronaridine–artesunate [Pyramax™; Shin-Poong Pharma Ltd., South Korea] first received a positive scientific opinion from the European Medicines Agency under Article 58 for the tablet formulation in 2012. Pyramax™ granules (a special paediatric formulation] were subsequently granted a positive scientific opinion through Article 58 in 2015 [15, 16]. This regulatory authority approval confirms that Pyramax™ can be used in malaria patients, particularly African children. The safety and efficacy of PA in adults and children in Africa and Asia have been demonstrated in many randomized controlled clinical trials [17–22]. Consequently, PA was recommended by the WHO in 2019 as a "safe and efficacious ACT for the treatment of uncomplicated malaria in adults and children weighing 5 kg or more in malaria-endemic areas" [23]. As a result, PA was included in Nigeria’s national treatment guidelines [13]. However, more research is needed to provide Nigeria with in-country data on the safety and efficacy of PA, based on the good safety and efficacy observed in the Phase III program and the CANTAM Study 2021 [24].

Furthermore, Sowunmi et al. [25] recently reported a decline in the responsiveness of childhood *P. falciparum* infections to ACT (both artemether–lumefantrine and artesunate-amodiaquine) in Nigeria. This underscores the importance of evaluating another effective and user-friendly anti-malarial with good efficacy and tolerability in the country. This clinical trial resulted from efforts to improve efficacy, tolerability, cost, and treatment regimens. As the country is endemic for malaria, it is also critical to continue assessing the efficacy of AL, the current first-line ACT of choice in Nigeria, in order to detect early loss of efficacy. There is currently no data on the safety and efficacy of PA in the Nigerian population. The goal of this study is to compare the safety and efficacy of PA and AL in children aged 3 months to 12 years (144 months) with symptomatic acute uncomplicated malaria in south-west Nigeria.

## Methods

### Study design and setting

This comparative, randomized, open-label, parallel-group clinical trial followed WHO guidelines [26]. Study drugs were PA granules or tablets (Pyramax™; 60 mg pyronaridine/20 mg artesunate per sachet of granules, 180/60 respectively per tablet). PA was supplied in aluminum sachets by Shin Poong Pharmaceutical Company, Ltd., Ansan, South Korea. Coartem™ (AL) dispersible tablets (Coartem™; Novartis SA, Basel, Switzerland) were obtained from the pharmaceutical representative of Novartis Pharma in Nigeria. AL was supplied in blister packs. Each tablet of dispersible Coartem contains 20 mg artemether and 120 mg lumefantrine. Patients were enrolled at the Kola Daisi Primary Healthcare Centre (PHC), University College Hospital (UCH), Ibadan, and Ikereku PHC in Akinyele Local Government Area, southwest Nigeria. The study was carried out from May 2019 to December 2019, and July 2020 to December 2020.

### Study participants

Children aged 3–144 months with clinical features consistent with acute uncomplicated malaria and microscopically confirmed *P. falciparum* malaria were enrolled if they had a minimum asexual parasite density of 1000/µL, an axillary temperature of ≥ 37.5 °C or a history of fever within 24 h of presentation as reported by a parent or caregiver, lived within fifteen kilometers of the study center, and could take drugs orally. Additional inclusion criteria included the absence of ACT intake in the 2 weeks preceding enrollment and signed informed consent from prospective enrollees’ parents or guardians to participate in the study. Children with a history of allergy to any of the study drugs, including artemisinin, and its derivatives, lumefantrine, or pyronaridine, were excluded from the study. Children with concurrent illnesses that could impair response evaluation, such as bacterial or viral infections, were also excluded. Additionally, children with clinical evidence of severe malaria were excluded from the study [27]. Children with severe malnutrition or known chronic diseases such as chronic liver disease, heart failure, or sickle cell anemia were also excluded from the study. Children of parents or guardians who in the investigator's opinion, will not adhere to the study protocol were not permitted to participate in the study. Research assistants were trained on strategies to retain participants in the study and track them by maintaining contact through scheduled home visits and telephone calls during the follow-up.

### Randomization and blinding

Patients were allocated to either PA or AL group following a pre-generated randomization table in ascending order. Different clinical personnel performed clinical assessments and drug administration.

### Treatments

The study drugs were administered orally for 3 days (days 0, 1, and 2). PA (Pyramax™—Shin Poong Pharmaceutical Company, Ltd., Ansan, Korea) was given once daily in the clinic by the research nurse supervised for 3 days. Pyramax™ contains 60 mg pyronaridine/20 mg artesunate per sachet of granules and 180 mg pyronaridine/60 mg artesunate per tablet supplied in aluminum sachets. Dosing was based on body weight: 5—< 8 kg: one sachet; 8—< 15 kg: two sachets; 15—< 20 kg; three sachets; 20—< 24 kg: one tablet and 24—< 45 kg: two tablets. All PA doses were administered with a milk drink under the supervision of a research nurse. AL was administered as dispersible tablets (Coartem™, Novartis Pharma Basil Switzerland). Each dispersible tablet contains 20 mg of artemether/120 mg of lumefantrine. Study participants were dosed as follows: 5—< 15 kg; one tablet, 15—< 25 kg two tablets, 25—< 35 kg three tablets, and 35 kg and above; four tablets. AL was administered at 0, 8, 24, 36, 48, and 60 h respectively. The first, third, and fifth doses of AL were administered supervised in the clinic with milk drinks while the second, fourth, and sixth doses of AL were administered with milk drinks at home by the parent or guardian. Mothers of children who received AL were encouraged to breastfeed very young babies who were still on the breast soon after the drug was administered. At the end of Days 0, 1, and 2 visits, parents and guardians were given a sachet of full cream powdered milk each daily for the 2nd, 4th, and 6th doses of AL and were also reminded by phone 30 min before the next drug dosing. Although a fatty meal was not required for optimal absorption of PA unlike for AL, all enrollees received study drugs with milk drinks because the children who were randomized to receive PA felt disadvantaged because they did not receive milk drinks, and this could lead to poor compliance with follow up visits.

Each enrollee irrespective of drug group was observed in the clinic for one hour after drug administration for vomiting. If vomiting occurred within 30 min of drug administration, the full treatment drug dose (PA or AL) was re-administered. If vomiting occurred between 30 and 60 min of drug administration, half the treatment dose was re-administered. Any enrollee who vomited the repeat dose was withdrawn from the study and treated with artesunate-amodiaquine (ASAQ) as rescue medication as per the Nigeria national malaria treatment guidelines [13].

### Data collection procedures

Enrollees who met the inclusion criteria were given a detailed explanation of the study and were only enrolled after the accompanying parent or guardian signed an informed consent form. The information gathered from each participant was entered into case record forms (CRF) created specifically for the study. In the individual CRF, socio-demographic information, contact information for each enrollee, past medical history, as well as clinical and laboratory details for all enrollees in the study, were recorded. Each enrollee’s parent or guardian provided a detailed medical history, which included a history of the presenting symptoms as well as a list of current medications. In 2019, an electronic thermometer was used to measure axillary temperature, while an infrared thermometer (beamed at the forehead) was used in 2020 because of the SARS-CoV-2 pandemic.

A few drops of capillary blood were obtained via finger prick for thick and thin blood smears and some into a capillary tube for hematocrit determination, and blood spots on filter paper for parasite genotyping at enrolment. For malaria parasite detection, thick and thin blood films were prepared from finger prick blood samples of each enrollee on Day 0, whereas only thick blood films were prepared at all other contact times and stained with fresh 10% Giemsa stain at pH 7.2. Dry stained blood films were viewed at a magnification of × 1000 using a light microscope in accordance with the WHO guidelines [26]. At enrolment and on all contact days, gametocyte carriage was specifically observed and counted in the thick films. At enrolment and at each follow-up visit, blood spots on filter paper were obtained for DNA analysis using polymerase chain reaction (PCR) to differentiate re-infection from recrudescence [28]. At each point of contact, blood was drawn into capillary tubes for hematocrit determination. A five-milliliter venous blood sample was also obtained via venipuncture for the evaluation of haematological and biochemical parameters (urea and creatinine, as well as a liver function test) on days 0, 3, 7, and 28. Blood samples were collected from study participants and analyzed at the chemical pathology and hematology laboratories of the University College Hospital Ibadan, Nigeria.

All enrollees were followed up on out-patient basis daily from days 0 to 3 and then on days 7, 14, 21 and 28. Enrollees were also seen whenever a participant fell ill or a parent or guardian was concerned about their children's health. The day before follow-up visits, parents and guardians of enrollees were reminded by phone. Enrollees were provided transportation refunds to encourage compliance with follow-up. Evaluation at each visit included a brief clinical history and a physical examination to assess new complaints and medication side effects. A finger prick blood sample was taken on days 0, 1, 2, 3, 7, 14, 21, 28, and any other (scheduled or unscheduled) visit day for the preparation of thick blood films for the identification and quantification of asexual stages of the malaria parasite while filter paper blood samples were collected on days 0, 3, 7, 14, 21, and 28 for parasite genotyping from the same capillary blood samples.

### DNA isolation and amplification of *Plasmodium falciparum*

Paired samples of blood spots on filter paper collected from patients with parasite recurrence on or before D28 (D0 and Day of parasite recurrence) were analyzed using nested PCR techniques to distinguish recrudescence from reinfection. Genomic DNA extraction was performed with QIAamp DNA Mini kit blood and tissue (QIAGEN Hilden Germany) kit, according to manufacturer’s instructions. Eluted genomic DNA was stored at − 20 °C until further PCR analysis. The small sub-unit ribosomal RNA (ssrRNA) gene of *P. falciparum* was amplified in a nested PCR using a primary genus-specific primer and a secondary *P. falciparum* species-specific primer following the previously described standard protocol [28–30]. Paired samples with confirmed parasitaemia were further genotyped to distinguish recrudescence from new infection using merozoite surface protein-1 (*msp-1*), merozoite surface protein-2 (*msp-2*) and glutamate-rich protein (*glurp*) genes, respectively.

In a 20 µL reaction volume, PCR mixture containing 5X PCR Master Mix (Solis Biodyne, Estonia), 0.5 mM each of forward and reverse primers, nuclease-free PCR grade water, and 5 µL of extracted genomic DNA as template was prepared for the primary reaction. In the Nested reaction, 2 µL of the primary PCR product was used as a template in 18 µL PCR mixture, containing the same reagents as the primary reaction except with a species-specific forward and reverse primers. The PCR thermal cycling conditions were previously described [29]. All amplification of DNA was performed in an Eppendorf AG 22331 thermal cycler (Hamburg, Germany).

### Genotyping of *msp-1, msp-2 *and* glurp* antigenic loci

PCR amplification targeting the polymorphic regions of *msp-1* (block 2), *msp-2* (block 3) and *glurp* (R2) regions were genotyped with allelic specific primers as previously described [30, 31]. The antigenic allelic families of *msp-1* (K1, RO33 and MAD20), *msp-2* (FC27 and 3D7) were amplified in a final reaction volume of 20 µL (25 µL for *glurp*), containing 5X PCR Master mix (Solis Biodyne, Estonia), primers (forward and reverse) 0.5 mM (0.75 mM for *glurp*) each and 5 µL of extracted genomic DNA as template in the primary reaction. Two µL of the primary PCR product was used as template in the nested reaction. The PCR cycling conditions for both primary and nested reactions were as previously described [30, 31]. Ten microliter (10 µL) of the nested PCR products (amplicon) were electrophoresed on 1.5% agarose gel pre-stained with EZ-vision blue light DNA dye (EZ-Vision^®^ Blue light DNA Dye, VWR Chemicals USA) and was allowed to migrate at 100 V for 40–60 min for fragment size differentiation. The gel was visualized and photographed under a UV trans-illuminator documentation system (UVP^®^ DigiDoc-It™, USA). Amplified fragments were paired for base-pair sizing and comparison. Fragments with the same base pair sizes (paired samples) in the amplified loci were considered recrudescence while un-identical base pair sizes were considered new infections. Alleles in each family were deemed to be identical if their fragment sizes fell within 20 and 50 base pair intervals, respectively [30, 31]. Fragments that are within the limits of this bin were considered as having identical genotype. Further details are shown on Table 1 in the Additional file 2.

**Table 1:** Baseline characteristics of children suffering from acute uncomplicated malaria treated with artemether–lumefantrine or pyronaridine–artesunate in Ibadan SW Nigeria

Characteristics	Drug group		Total	*ρ*-value
	AL	PA		
Sex				
Male	45 (50.0%)	45 (50.0)	90 (100)	0.880
Female	42 (51.2%)	40 (48.8%)	82 (100)	
Age (months)				
Mean ± SD	79.93 ± 37.16	82.39 ± 38.63	81.15 ± 39.80	0.671
Range	8–144	4–143	4–144	
3–11 months	3 (3.5)	5 (5.9)	8 (4.7)	0.703
12–59 months	20 (23.0)	21 (24.7)	41 (23.8)	
≥ 60 months	64 (73.6)	59 (69.4)	123 (71.5)	
Weight (kg)				
Mean ± SD	18.59 ± 6.49	18.67 ± 6.74	18.63 ± 6.59	0.938
Range	7.0–36.0	5.5–40.0	5.5–40	
Temperature (°C)				
Mean ± SD	37.7 ± 1.06	37.4 ± 1.07	37.6 ± 1.08	0.074
Range	35.1–40.2	35.9–39.9	35.1–40.2	
Parasite density (/µl)				
Geomean	32,755	28,211	30,424	0.815
Range	1035–758,958	1600–1,652,505	1035–1,652,505	
Haematocrit (%)				
Mean ± SD	32.14 ± 5.06	32.14 ± 5.48	32.14 ± 5.26	0.997
Range	18–41	18–48	18–48	
Anaemic at DO				
Haematocrit < 30%)	19 (21.8)	21 (24.7)	40 (23.3)	0.396
Height (cm)				
Mean ± SD	110.65 ± 21.31	110.65 ± 28.90	110.38 ± 25.36	0.892
Range	18–143	18–186	18–186	

Paired samples of blood spots on filter paper collected from patients with parasite recurrence on or before D28 (D0 and Day of parasite recurrence) were analyzed using PCR techniques with merozoites surface protein 1 (msp1), merozoites surface protein 2 (msp2) and glutamate-rich protein (glurp) according to WHO global guidance on distinguishing recrudescence from re-infection [1, 2]. The global guidance using WHO/World-Wide Antimalaria Resistance Network (WWARN) alternative approach termed the 2/3 algorithm, whereby *msp1/msp2* are evaluated, and only in cases where these two markers are discordant, *glurp* is used as the deciding factor. In this case, even if *msp1* indicates a reinfection, if *msp2* and *glurp* indicate a recrudescence, the recurrence is deemed a recrudescence. Reinfection was defined as when a follow up sample contained only new alleles that are un-identical with day 0 sample.

### Study endpoints

The WHO 2009 treatment outcome criteria were used to assess efficacy [27]. Early treatment failure (ETF) was defined as danger signs or severe malaria on day 1, 2 or 3 in the presence of parasitaemia; parasitaemia on day 2 higher than on day 0, irrespective of axillary temperature; parasitaemia on day 3 with an axillary temperature ≥ 37.5 °C or parasitaemia on day 3 ≥ 25% of count on day 0. Late clinical failure (LCF) was defined as danger signs or severe malaria in the presence of parasitaemia on any day between day 4 and day 28 (day 42) in patients who had not previously met any of the criteria for early treatment failure, or the presence of parasitaemia on any day between day 4 and day 28 (day 42) with an axillary temperature of ≥ 37.5 °C (or history of fever) in patients who had not previously met any of the criteria for early treatment failure. Late parasitological failure (LPF) was defined as the presence of parasitaemia on any day between days 7 and 28 (day 42) and an axillary temperature of < 37.5 °C in patients who did not previously meet any of the criteria for early treatment failure or late clinical failure. The main end point for the study was adequate clinical and parasitological response (ACPR), defined as the absence of parasitaemia on day 28, irrespective of axillary temperature, in patients who had not previously met any of the criteria of early treatment failure, late clinical failure, or late parasitological failure.

Recrudescence was defined as the recurrence of asexual parasitaemia within 28 days of receiving anti-malarial treatment using the 2021 WHO 2/3 algorithm as stated above for both Day 0 and Day of failure blood spot samples Parasite clearance time (PCT) was defined as the time from the first dose of ACT until the first total and continued disappearance of asexual parasite forms for at least 24 h, whereas fever clearance time (FCT) was defined as the time from the first dose of ACT until the first time the body temperature (for those with a raised temperature at enrolment) drops to below 37.5 °C and stays below 37.5 °C for at least 24 h.

During this study, safety was evaluated in terms of adverse events and severe adverse events by examining symptoms, clinical signs, and laboratory parameters. All study participants who took at least one dose of either of the study drugs were evaluated for safety. The evaluation of liver enzymes was a special category that was evaluated due to previous reports of transaminitis [22]. Alanine transaminase (ALT)/aspartate transaminase (AST) greater than three times the upper limit of normal (ULN) plus peak total bilirubin greater than two times the ULN in the absence of a significant alkaline phosphatase increase were considered serious adverse events (SAE). Any ALT and AST level greater than 5 times the ULN were considered an adverse event of special interest (AESI).

### Sample size calculation

The required minimum number of enrollees was calculated using the sample size formula for the test of non-inferiority as written by Chow and colleagues [33]. By assuming a difference of 2.5% in cure rate between PA and AL (98.9% vs. 96.4%), 140 patients are required to attain a power of 90% at a 95% level of confidence to demonstrate non-inferiority of PA versus AL with a non-inferiority limit of 5%. However, allowing for 20% loss in follow-up, the total number of patients required would be 168, giving 84 patients per arm.

### Data analysis

All study participants who received any amount of the study drugs were included in the intent-to-treat population. The per-protocol-population included children who received the entire course of study medication, had a known day-28 efficacy end point, and had no major protocol deviations. The data was analyzed for efficacy in both the intention-to-treat and per-protocol populations. The efficacy endpoints were summarized by age. Safety outcomes were adverse events and abnormalities of laboratory results including liver function test and haematological indices. SPSS IBM Statistic Software version 20 was used to analyze the data at *ρ* =  < 0.05 and adjustment was made for multiple comparisons with the Bonferroni correction.

## Results

### Enrollees’ baseline characteristics and symptoms at presentation

**Fig. 1:**
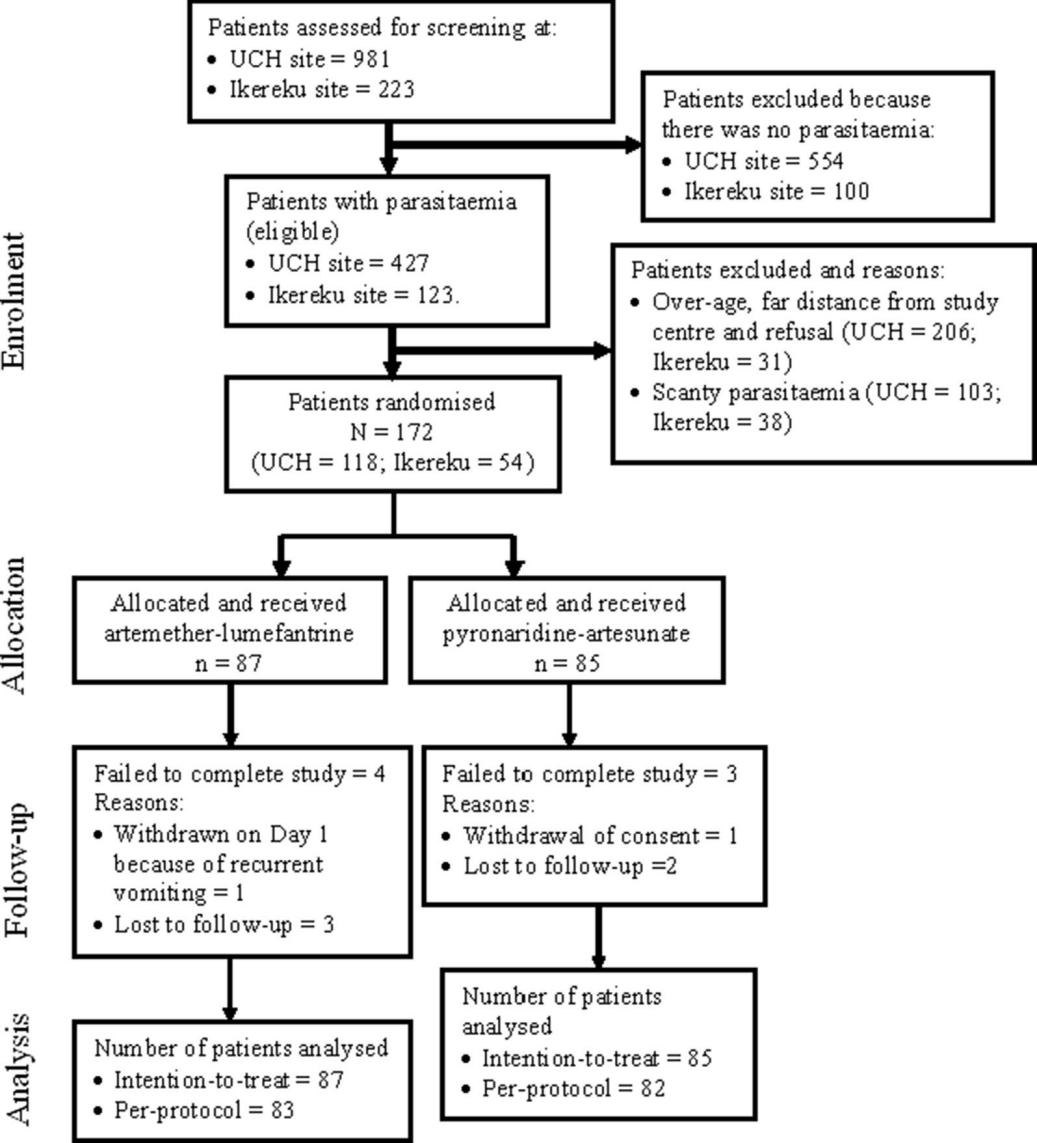
Study design and patient flow among children from southwest Nigeria with acute uncomplicated malaria treated with artemether–lumefantrine or pyronaridine–artesunate

The study was conducted between May and December 2019 and between July and December 2020. A total of 1204 febrile children suspected of having malaria were screened for malaria parasitaemia during the study period; 550 (45.7%) had patent parasitaemia, with 172 eligible patients randomly assigned to PA (n = 85) and to AL (n = 87). Figure 1: study design and patient flow, shows the details of the reasons for the exclusion of ineligible patients.

The four most common symptoms at presentation were fever (100%), headache (69.8%), chills and rigors (62.6%), and loss of appetite (57.2%) (Table 2). Table 2 also shows that there were statistically significant differences in the frequencies of only chills and rigors and loss of appetite which were higher in the AL group than in the PA group (*ρ* = 0.033 and 0.014, respectively). Five patients (5.9%) in the PA compared with 3 (3.4%) in the AL group (*ρ* = 0.347), vomited the first dose of the study drug and had to be re-dosed. After redosing, one of the children that received AL vomited and was withdrawn from the study.

**Table 2:** Distribution of children with acute uncomplicated malaria according to symptoms other than fever at presentation by treatment groups

Presenting complaint	Total population	Artemether–lumefantrine	Pyronaridine–artesunate	*ρ*-value
	N = 172 (100)	N = 87 (100%)	N = 85 (100%)	
Headaches	120 (69.8)	61 (70.1)	59 (69.4)	1.000
Chills and rigors	117 (68.0)	66 (75.9)	51 (60)	0.033
Loss of appetite	101 (58.7)	59 (67.8)	42 (49.4)	0.014
Vomiting	82 (47.7)	44 (50.6)	38 (44.7)	0.450
Abdominal pain	68 (39.5)	33 (37.9)	35 (41.7)	0.642
Nausea	54 (31.4)	30 (34.5)	24 (28.6)	0.417
Cough	45 (26.2)	21 (24.1)	24 (28.6)	0.603
Diarrhea	32 (18.6)	18 (20.7)	14 (16.5)	0.558
Irritability	33 (19.2)	13 (14.9)	20 (23.5)	0.178
Insomnia	17 (9.9)	10 (11.5)	7 (8.3)	0.611
Palpitations	10 (5.8)	5 (6.0)	5 (5.7)	1.000

### Results of efficacy evaluation

#### Intention-to-treat population analysis

Of the 172 participants randomized to the two study groups, 165 (95.9%) completed the study, and the primary efficacy endpoint of the study was attained. The distribution of the participants in the two study groups by treatment outcomes based on intention-to-treat and per-protocol analyses is shown in Table 3. There were no significant differences in the proportion of participants who completed the study (*p* = 0.983), those who were lost to follow-up (*p* = 0.487) and those who withdrew/were withdrawn (*p* = 0.979) between the two study groups.

**Table 3:** Treatment outcome among patients with uncomplicated malaria treated with Artemether–lumefantrine or Pyronaridine–artesunate in Ibadan, southwest Nigeria

Treatment outcome	Artemether–lumefantrine	Pyronaridine–artesunate	Total	*ρ*-value
	N (%)	N (%)	N (%)	
TOTAL enrolled	87 (100)	85 (100)	172 (100)	
Completed study	83 (95.4)	82 (96.5)	165 (95.9)	0.535
LTFU	3 (3.4)	2 (2.4)	5 (2.9)	
Withdrawn	1 (1.2)	0 (0.0)	1 (0.6)	
Withdrawal of consent	0 (0.0)	1 (1.2)	1 (0.6)	
ITT-uncorrected	N = 87 (100)	N = 85 (100)	N = 172 (100)	
ACPR—D28	59 (67.8)	76 (89.4)	135 (78.5)	0.001
Treatment failure	24 (27.6)	6 (7.1)	30 (17.4)	
LTFU/withdrawn	4 (4.6)	3 (3.5)	7 (4.1)	
PP—uncorrected	N = 83	N = 82	N = 165	
ACPR—D21	73/83 (88.0)	82/83 (98.8)	135/166 (93.4)	0.016
ACPR—D28	59/83 (71.1)	76/82 (92.7)	135/165 (81.8)	
LPF—D28	17 (20.5)	6 (7.3)	23 (13.9)	0.001
LCF—D28	7 (8.4)	0 (0.0)	7 (4.2)	
PP-PCR corrected	N = 67	N = 78	N = 145	
Cure rate—D28	59 (88.1)	76 (97.4)	135 (93.1	0.04
Recrudescence	8 (11.9)	2 (2.6)	9 (6.9)	
Day of failure [N (%)]	24 (110)	6 (100)	30 (100)	
Day 14	2 (8.3)	0 (0.0)	2 (6.7)	
Day 21	9 (37.5)	1 (16.7)	10 (33.3)	0.405
Day 28	13 (54.2)	5 (83.3)	18 (60.0)	
% Failed before D28	11 (45.8)	1 (16.7)	12 (40.0)	
% Failed on D28	13 (54.2)	5 (83.3)	18 (60.0)	0.358
Parasite clearance time (days)				
Mean ± SD	2.15 ± 0.68	2.08 ± 0.64	2.12 ± 0.66	0.430
Range	1–4	1–3	1–4	
Fever clearance time (days)				
Mean ± SD	1.11 ± 0.32	1.13 ± 0.44	1.13 ± 0.38	0.664
Range	1–2	1–3	1–3	
Haematocrit at D28 (in %)				
Mean ± SD	33.3 ± 2.95	34.9 ± 2.79	34.13 ± 2.95	0.002
Range	23–40	28–42	25–42	
Haematocrit < 30% D28	1 (1.4)	1 (1.4)	2 (1.4)	1.000

*PP* per-protocol population, *ITT* intent-to-treat population, *ACPR* adequate clinical and parasitological cure, *LTF* late treatment failure, *LCF* late clinical failure, *LTFU* lost to follow up

Among the intention-to-treat population, the percentage of patients with adequate clinical and parasitological responses (ACPR) on day-28 was significantly higher among patients in the PA group than the AL group (*ρ* =  < 0.001) (Table 3). The total number of patients who were considered to have experienced treatment failure in the AL group [parasite recurrence (24) + LTFU (3) + withdrawal (1) = 28/87 (32.2%)] was significantly higher than those who were considered to have had treatment failure in the PA group [parasite recurrence (6) + LTFU (2) + withdrawal (1) = 9/85 (10.6%)]. The risk of failure was also higher in the AL group than in the PA group (RR = 1.80; 95% CI 1.39, 2.33), *ρ* =  < 0.001. Similarly, patients randomized to the AL group had an increased risk of experiencing treatment failure before day-21 compared with those in the PA group (RR = 1.87; 95% CI 1.44, 2.43).

In the study being reported here, there were 13 enrollees who had parasite density > 200,000/μL, 6 received AL while 7 received PA. The ages of the children with parasite density > 200,000/µL ranged from 40 to 132 months. None of the 13 had evidence of any organ failure. One was lost to follow up after Day 21. The enrollee lost to follow up, was free of patent parasitaemia when last seen on Day 21. Nine of the remaining 12 recorded ACPR at day 28 (PA = 5, AL = 4) while three (PA = 1; AL = 2) recorded late parasite failure, one on Day 21 (AL treated enrollee) and the other two (PA = 1; AL = 1) on Day 28.

**Fig. 2:**
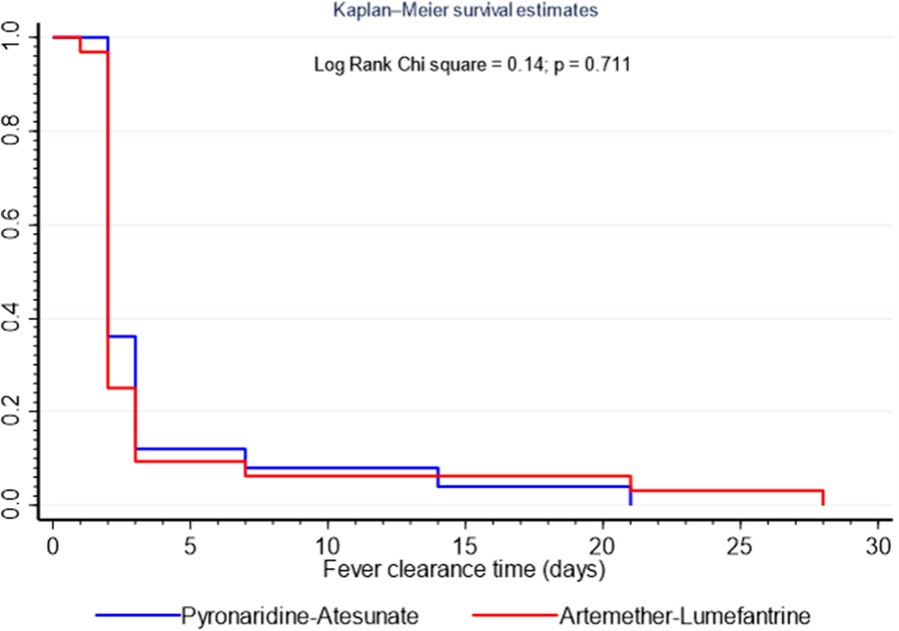
Kaplan–Meier analysis (intent-to-treat population) comparing fever clearance time (FCT) between pyronaridine–artesunate and artemether–lumefantrine

**Fig. 3:**
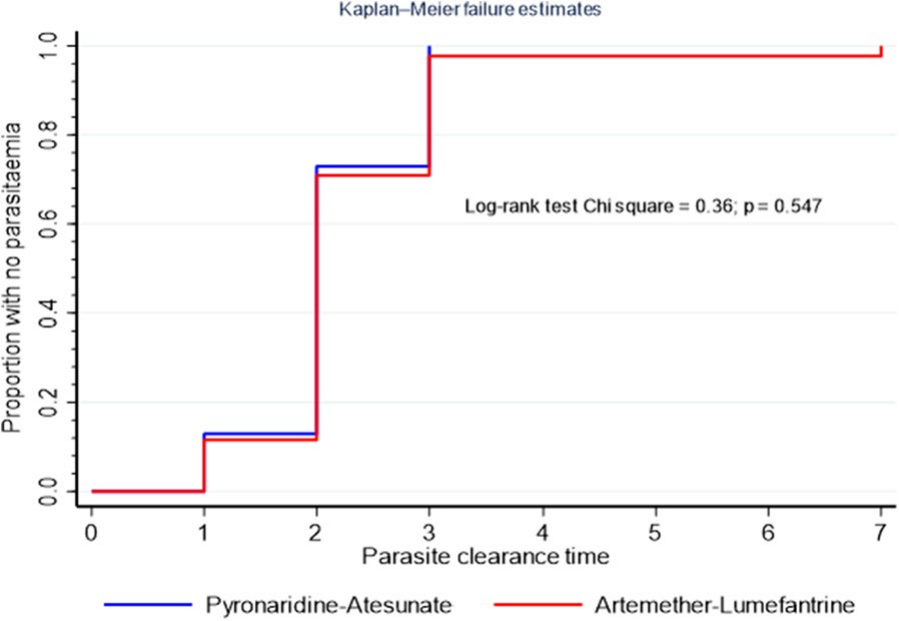
Kaplan–Meier analysis (intent-to-treat population) comparing parasite clearance time (PCT) between pyronaridine–artesunate and artemether–lumefantrine

Although haematological recovery was excellent for both study drugs, the mean hematocrit on day 28 was significantly higher in the patients randomized to the PA group (34.9 ± 2.8%) than in those in the AL group (33.3 ± 3.0%); *ρ* < 0.002. Conversely, there were no significant differences in the mean parasite and fever clearance times as well as the number of anaemic patients between the PA group and the AL group (Table 3). The graphs (Figs. 2, 3) from the Kaplan–Meier survival analysis also corroborate the lack of differences in the fever and parasite clearance times between patients randomized to the PA group and those in the AL group.

#### Per-protocol population analysis

There was no case of early treatment failure during the study. Response to treatment was prompt in all study participants except in an enrollee among AL treated children who had recurrent vomiting and had to be withdrawn on day 0. By day 1, 11.6% (10/86) and 12.9% (n = 11/85) of patients treated with AL and PA respectively were free of patent parasitaemia while corresponding values for day 2 were 70.9% (n = 61/86) and 72.9% (n = 62/85) for AL and PA, respectively. Two (2.3%) of the study participants who received AL had patent parasitaemia at day 3, but they both recorded adequate clinical and parasitological responses at day 28. None of those treated with PA had patent parasitaemia on Day 3. All participants were free of patent parasitaemia by day 7. However, two children among those who received AL failed treatment at day 14, one each recorded LCF and LPF.

In contrast to those treated with AL, treatment failure was first seen among children who received PA on day 21. The ACPR in the per-protocol population at day 21 was significantly higher for PA than for AL treated children (98.8% vs. 88.0%, respectively, *ρ* = 0.009). The chances of ACPR were significantly lower in the AL than PA (RR = 0.52, 95% CI 0.40, 0.67) treated children.

The cumulative incidence of ACPR over 28 days among PA treated children without PCR-correction and PCR-corrected were 92.7% (95% CI (84.6–96.7) and 97.4% (95% CI 90.8–99.4), respectively. For AL treated children, the cumulative incidence of ACPR over 28 days without PCR-correction and PCR-corrected were 71.1% (95% CI 60.3–79.8) and 88.1% (95% CI 80.9–94.9). In calculating the PCR corrected cure rates, the cases of reinfection were censored and as such were not included in the numerator or denominator.

The efficacy outcomes observed by age in the per-protocol population are as shown in Table 4. The ACPR was significantly higher in the PA group than in the AL group (*ρ* = 0.008) among children under 5-year-old (< 60 months) and over 5-year-old (≥ 60 months) children (RR = 1.64; 95% CI 1.19, 2.26), *ρ* = 0.016, like what was recorded in the entire study population.

**Table 4:** Treatment outcome among children (PP) from southwest Nigeria with acute uncomplicated malaria treated with artemether–lumefantrine or pyronaridine–artesunate in different age groups

Treatment outcome	Drug group		Total	*ρ*-value
	AL	PA		
Age < 12 months (N = 8)	3	5	8	
ACPR	2 (66.7)	5 (100)	7 (87.5)	0.375
LCF	1 (33.3)	0 (0)	1 (12.5)	
Age < 60 months (N = 47)	N = 22	N = 25	N = 47	
ACPR	14 (63.6)	24 (96.0)	38 (80.9)	0.008
LPF	4 (18.2)	1 (4.0)	5 (10.68)	
LCF	4 (18.2)	0 (0)	4 (8.5)	
Age ≥ 60 months (N = 118)	N = 61	N = 57	N = 118	
Cured (ACPR)	45 (73.8)	52 (91.2)	97 (82.2)	0.016
Failed (LCF + LPF)	16 (26.2)	5 (6.1)	21 (17.8)	
ACPR	45 (73.8)	52 (91.2)	97 (82.2)	0.046
LPF	13 (21.3)	4 (7.0)	17 (14.4)	
LCF	3 (4.9)	1 (1.8)	4 (3.4)	
Total PP (N = 165)	83	82	165	
ACPR	59/83 (71.1)	76/82 (92.7)	135/165 (81.8)	0.001
LPF	17/83 (20.5)	5/82 (6.1)	22 (13.3)	
LCF	7/83 (8.4)	1/82 (1.2)	8 (4.8)	
Cured (ACPR)	59/83 (71.1)	76/82 (92.7)	135/165 (81.8)	< 0.0001
Failed (LCF + LPF)	24/83 (28.9)	6/82 (7.3)	30/165 (18.2)	
PP-PCR corrected	N = 67	N = 78	N = 145	0.04
Cure rate—D28	59 (88.1)	76 (97.4)	135 (93.1)	
Recrudescence	8 (11.9)	2 (02.6)	10 (6.9)	

All the 30 children with parasite recurrence among per-protocol (PP) population—[6/82 (7.3%) for PA vs. 24/83 (28.9%) for AL; *ρ* < 0.001] were treated with artesunate-amodiaquine (ASAQ). Response to ASAQ was prompt, leading to rapid parasite clearance and resolution of symptoms.

### Molecular analysis

Molecular biology analysis of paired samples for enrollees with parasite recurrence was carried out using *msp-1, msp-*2 and *glurp*. This showed that four of the six cases of parasite recurrence among children treated with PA were new infections while two were recrudescence. Sixteen of the 24 cases of treatment failure among AL treated children were also new infections while eight were recrudescence giving PCR corrected cure rates of 97.4% (76/78) for PA and 88.1% (59/67) for AL for the PP populations. To arrive at these cure rates, new infections, were censored which led to denominators of 78 (82–4) for PA group and 67 (83–16) for AL treated group.

**Fig. 4:**
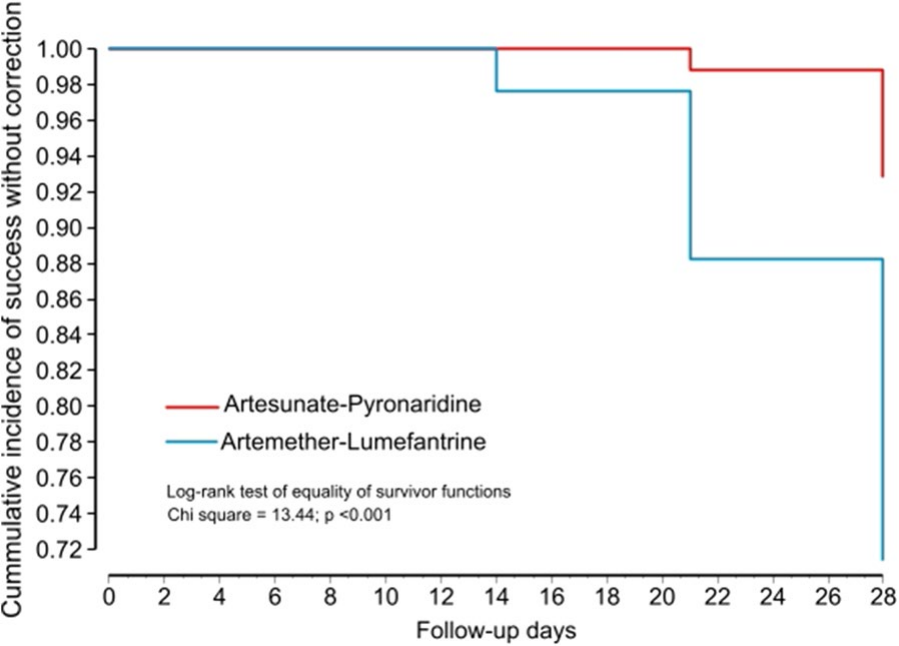
Kaplan–Meier survival analysis comparing the ACPR over 28 days between pyronaridine–artesunate and artemether–lumefantrine without PCR correction

**Fig. 5:**
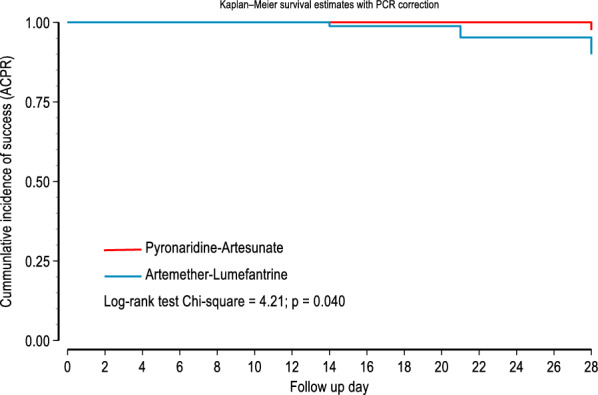
Kaplan–Meier survival analysis comparing the ACPR over 28 days between pyronaridine–artesunate and artemether–lumefantrine with PCR correction after treatment

The Kaplan–Meier survival estimates (Fig. 4) showed that the cumulative incidence of ACPR day 28 in the Pyronaridine–Artesunate-group were significantly higher than the chances of survival in the Artemether–Lumefantrine group without PCR correction (*p* < 0.001) and with PCR correction (*p* = 0.040) (Fig. 5).

### Safety of pyronaridine–artesunate compared with artemether–lumefantrine

#### Complaints and physical examination

The ITT population was considered as the safety population. Recorded adverse events were similar to the symptoms and signs seen during malaria infection. There was no incidence of serious adverse event throughout the study. The type and prevalence of the various adverse events were similar for the two study drugs. The five most often recorded adverse events were fever, chills and rigors, anorexia, cough, and headache. These are clinical symptoms that occur during malaria infection. Further details are provided in Table 5. There was no clinical evidence of jaundice, intravascular hemolysis, hepatic dysfunction, or renal impairment. No death was recorded during the study.

**Table 5:** Summary of adverse events among children in the per-protocol population (PP) from southwest Nigeria with acute uncomplicated malaria treated with artemether–lumefantrine or pyronaridine–artesunate

Event	All patients	Artemether–lumefantrine	Pyronaridine–artesunate	*p*
	N = 172	N = 87	N = 85	
Adverse event from any cause	53 (30.8)	27 (31.0)	26 (30.6)	0.949
Fever	58 (34.1)	24 (27.9)	34 (40.5)	0.106
Chills and rigors	33 (19.6)	16 (18.6)	17 (20.5)	0.847
Anorexia	29 (17.1)	16 (18.6)	13 (15.5)	0.685
Cough	28 (16.6)	14 (16.3)	14 (16.9)	1.000
Headache	27 (16.0)	12 (14.1)	15 (17.9)	0.536
Fatigue	22 (12.9)	11 (12.6)	11 (13.1)	1.000
Pallor	17 (10.1)	9 (10.6)	8 (9.6)	1.000
Abdominal pains	12 (7.1)	5 (5.8)	7 (8.3)	0.563
Vomiting	11 (6.4)	6 (7.0)	5 (5.9)	1.000
Diarrhea	11 (6.4)	4 (4.6)	5 (6.0)	0.744
Palpitations	6 (3.5)	3 (3.4)	3 (3.6)	1.000
Nausea	6 (3.5)	3 (3.5)	3 (3.6)	1.000
Rashes	1 (0.6)	0 (0.00)	1 (1.2)	1.000

*AL* artemether–lumefantrine, *PA* pyronaridine–artesunate

*Chi square was used but with Yate correction where cell counts were less than 5

Laboratory results were mostly within normal ranges, with occasional marginal increases as (shown in Additional file 1: Tables S1a–S1h, Tables S2a–S2h and Figs. S1–S8). There were no records of blood urea, creatinine, random blood glucose, or total bilirubin levels that were more than twice the upper limit of normal after receiving either study drugs. Both PA and AL had no deleterious effect on the transaminases. On day-0 (before anti-malarial administration), two study participants who received AL had alanine transaminase (ALT) levels that were twice the upper limit of normal. By day 3, the elevated ALT levels had returned to normal. On day 0, one of the two children who received AL and had ALT more than twice the upper limit of normal also had aspartate aminotransferase (AST) more than twice the upper limit of normal, which returned to normal by day 3.

On day-3, one participant in the PA group had AST level that was twice the upper limit of normal. By day 7, the elevated AST level had returned to normal. There was no instance of any adverse event of interest. Changes in blood levels of various enzymes and other parameters measured among participants in the two drug groups were statistically significant on Days 7 and 28 with regards to ALT and Day 28 with AST and alkaline phosphatase (ALP). The observed increases in liver enzymes, bilirubin, urea, creatinine, and random blood sugar levels were not clinically significant (Additional file 1).

### Gametocyte carriage

The number of participants who received PA with gametocyte carriage was consistently higher than that of those who received AL, except on day 28, when only one of the PA-treated children and two of the AL-treated children had gametocytes. However, the differences were only statistically significant on day 7. Additional file 2: Table S3a contains further information on gametocyte carriage. The number of participants with gametocytes was not only higher in children treated with PA, but the gametocyte densities were also consistently higher (Additional file 2: Table S3b). One participant in particular stood out because he had gametocytes from day 0 to day 28. The gametocyte counts were also high, ranging from 728/µL to 3088/µL. Two other participants did not clear their gametocytes until day 21, and another participant had gametocytemia until day 14. All four participants who had delayed gametocyte clearance had received PA.

## Discussion

During this study, the comparative efficacy and safety of PA and AL in the treatment of acute uncomplicated falciparum malaria among Nigerian children aged 3–144 months with parasite densities that ranged from 1035/µL to 1,652,505/µL were evaluated. Even though children with parasite density above 200,000/µL, were enrolled into the study, no clinical case of severe malaria was enrolled as none of the children enrolled into the study had their malaria infection complicated by serious organ failures or abnormalities in the patient's blood or metabolism. Children who live in malaria endemic regions, like Nigeria, frequently present to malaria clinic with parasite densities > 200,000/µL and are successfully treated with oral anti-malarial medications as outpatients [34, 35]. This is unlike the situation in areas of the world where malaria is hypo-endemic or areas of unstable malaria transmission [36]. Outside of the therapeutic efficacy study or other protocol design confines, it is critical that evaluation of the efficacy of anti-malarial drugs in malaria endemic countries like Nigeria covers the range of parasitaemia that healthcare workers in the environment will encounter on a daily basis. However, only a small number of healthcare workers in Nigeria have access to quality assured malaria microscopy, while malaria rapid diagnostic tests, a qualitative test recommended for programmatic deployment by Nigeria's national malaria elimination program [13], are more widely available.

The trial participants tolerated the two artemisinin-based combinations satisfactorily. There was no record of early treatment failure in either arm of the study, and only one participant who received AL was withdrawn as a result of recurrent vomiting. PA was found to be significantly more efficacious than AL among both the intent-to-treat (89.4% vs. 67.8%, *ρ* = 0.001) population, and the per-protocol population (92.7% vs. 71.1%; *ρ* = 0.001) without PCR correction. Details can be seen in Table 3. The primary efficacy outcome for PA was also greater than 90% in all age groups (Table 4), be it among children under 1 year (< 12 months) of age, under-5 years old (< 60 months), or 60–144 months old. The efficacy of PA against falciparum malaria was generally consistent with previous studies on the use of PA [17–24, 37, 38].

A highly significant efficacy finding in this study is that PA had a statistically significantly higher ACPR than AL in both the intent-to-treat (89.4% vs. 67.8%; *ρ* = 0.001) and per-protocol populations (92.7% vs. 71.1%; *ρ* = 0.001), as shown on Table 3. Among the PP population, sixteen of 24 (66.7%) and eight of 24 (33.3%) cases of parasite recurrence by Day 28 among AL treated children were new infections and recrudescence, respectively. This contrasts with the findings among PA-treated children, in whom only 6 children had parasite recurrence, of which 4 and 2, respectively, were new infections and recrudescence. This finding raises concern about the post treatment prophylaxis of AL compared with PA. However, the proportions were the same at (4/6) 66.7% re-infection and (2/6) 33.3% recrudescence, respectively, for PA.

The day-28 PCR-corrected cure rate among the children with uncomplicated falciparum malaria treated with PA granules or tablets was 97.4% (76/78) in the per-protocol population, while the PCR-corrected cure rate among AL treated enrollees was 88.1% (59/67) after censoring cases with reinfections. The Kaplan–Meier survival estimates also showed that the cumulative incidence of ACPR over the 28 days of follow-up in the Pyronaridine-Artesunate group was significantly higher than that in the Artemether–Lumefantrine group without PCR correction (*p* < 0.001) and with PCR correction (*p* = 0.040). It is worrisome that 28.9% [24/83] of AL-treated enrollees had parasite recurrence by day 28, irrespective of whether it was a reinfection or recrudescence. This is far from ideal for the children who go through another episode of malaria infection at such short intervals, with all the health and socio-economic consequences for them and their families. The recorded high efficacy of PA is in keeping with reports of PA efficacy in the treatment of acute uncomplicated malaria among Gabonese children [38], Kenyan children [37], and children from Burkina Faso, Guinea, and Mali [21]. Although there are no reports on the efficacy and safety of PA in Nigeria, numerous studies have reported on the efficacy of AL, the comparator drug during this study, in the management of acute uncomplicated malaria in Africa in general and Nigeria in particular [39–42].

It is particularly noteworthy that PA demonstrated a statistically significant higher PCR uncorrected efficacy among under 5-year-olds during this study [96.0% vs. 63.6% for PA and AL, respectively, as shown in Table 4 (*ρ* = 0.008). PA also recorded a statistically significantly higher efficacy among children ≥ 60 months than AL [91.2% vs. 73.8% for PA vs. AL; *ρ* = 0.016]. Details are shown in Table 4. It can be assumed that PA’s significant superiority among children ≤ 60 months of age reflects its true efficacy since this group of children is generally believed to be relatively immuno-naïve and the chemotherapeutic effect of AL was not augmented by immunity.

There was no difference in parasite clearance time between PA and AL treated groups, and the findings during this study are consistent with Roth and colleagues' findings among Kenyan children [37]. In contrast, three previous studies demonstrated that PA group cleared *P. falciparum* more rapidly than AL [17, 20]. On day 3, all enrollees were fever-free, and no difference in fever clearance time was observed, which is consistent with a previous study [20], though another study found that PA caused faster fever clearance [17]. The occurrence of one LCF and one LPF on D14 among participants who received AL is worrisome. This is most likely another indication of declining efficacy of AL in the treatment of malaria in the study area. The Day-28 ACPR of 71.1% during this study is further confirmation of declining efficacy of AL in the treatment of malaria among Nigerian children. Sowunmi et al*.* [25] previously reported a decline in the responsiveness of uncomplicated malaria to Nigeria's preferred anti-malarial drugs—AL and ASAQ. This was stated in a report detailing a post-hoc analysis of clinical trials in south-west of Nigeria among 360 children under 16 years at 5-year intervals in 2009–2010, and 2014–2015, and 1341 children under 5 years in studies from six-geopolitical zones from the Nigerian national anti-malarial therapeutic efficacy studies conducted at 2-year intervals in 2009–2010 and 2012–2015, respectively, after deployment in 2005. It is of great concern that the uncorrected and PCR-corrected Day-28 cure rates of AL (71.1% and 88.1% respectively) have fallen below the WHO recommended cut off level > 10% anti-malarial treatment failure with regards to making a policy change of first-line anti-malarial [43]. This is clearly a red flag to the National Malaria Elimination Programme (NMEP).

In this study, both AL and PA had good safety profiles. The commonly observed adverse events were fever, chills, rigors, anorexia, and headache. These symptoms are like those seen in malaria patients and resolve as the infection is cleared. These findings are consistent with those reported by earlier researchers [20, 22, 24, 38, 40]. There was no record of clinical hepatic impairment for PA or AL. Laboratory evaluation of transaminases, alkaline phosphatase, and bilirubin only revealed marginal increases with very few records of twice the upper limit of normal in both treatment arms (Additional file 1: Tables S1a–S1h, Tables S2a–S2h, and Figs. S1–S8). The alterations in transaminases were similar in both treatment arms and were not clinically significant. This contrasts with the findings of Sagara et al. [44], who reported that 13 out of 996 (1.3%) patients had transaminases increase after the first cure, including one possible Hy's law case, and two out of 311 (0.32%) after a retreatment of a second bout of malaria with PA. The lack of any cases of significant transaminase increases could be attributed to the relatively small number of participants (85) in this study compared to 996 in the study reported by Tshefu et al. [20]. Our observation is, however, supported by the report of Lutete et al. [24] in their cohort event monitoring study in five African countries involving over 7000 malaria patients in a protocol resembling real-world clinical practice.

Another notable finding from the present study is that from days 0 to 21, gametocyte carriage was consistently higher among PA-treated children compared to AL-treated children, but the observed differences were not statistically significant (Additional file 2: Tables S3a and S3b). The significance of this relatively higher gametocyte carriage is unclear, as the presence of gametocytes does not always indicate infectivity [44–47]. Gametocytes are the stage in the plasmodial life cycle that is infectious to mosquitoes, the insect in which sexual multiplication of malaria parasites occurs, leading to the development of sporozoites, which in turn infect humans. However, only mature gametocytes are infectious to mosquitoes after ingestion. Furthermore, microscopy cannot distinguish between viable living gametocytes and dead or drug-affected gametocytes. Nonetheless, the high gametocyte carriage suggests that pyronaridine does not have particularly effective gametocidal properties. The pyronaridine nucleus is derived from mepacrine (9-aminoacridine) and contains an amodiaquine-like side chain. In a systematic review, the WWARN study group reported clear differences in gametocyte clearance between various artemisinin-based combinations [48]. The WWARN study group reported that ACTs with 4-aminoquinoline partner drugs, such as dihydroartemisinin-piperaquine and artesunate amodiaquine, clear gametocytes at a much slower rate than those with aryl-amino alcohol and related structures (artesunate-mefloquine and artemether–lumefantrine).

This study's findings should be viewed with a few caveats in mind. The current study included only eight children under the age of 12 months, of which only five were randomly assigned to the pyronaridine–artesunate group. Even though the ACPR for the PA group was 100 percent on day 28, the number of enrollees is insufficient to draw any conclusions about efficacy and safety in that age group. As a result, more information on this patient population is needed for pyronaridine–artesunate.

## Conclusion

The efficacy and safety of PA among Nigerian children is both impressive and important as Nigeria is one of the highest burden countries for malaria in SSA especially as this is being recorded at a time that AL has started to show declining efficacy. This study provides empirical evidence for the inclusion of PA in the National Treatment Guidelines, and it is hoped that findings from routine TES will provide relevant updates on the efficacy of ACT in the country for their sustained recommendation.

## Supplementary Information

**Additional file 1**. **Table S1**: Paired sample genotyping data. **Tables S1a–S1h** and **Tables S2a–S2h **and **Figs. S1–S8 **of results showing liver enzymes, serum bilirubin, urea, creatinine, glucose and glucose of children enrolled in the study.

**Additional file 2.** **Table S3:** **Tables S3a** and **S3b **showing gametocyte carriage among children treated with artemether–lumefantrine and pyronaridine–artesunate during the study.

## Data Availability

The datasets used and/or analyzed during the current study are available from the corresponding author upon reasonable request.
