# Supplementary material for: Efficacy and safety of pyronaridine–artesunate versus artemether–lumefantrine in the treatment of acute uncomplicated malaria in children in South-West Nigeria: an open-labelled randomized controlled trial
Source: Malar J. 2023 May 13;22:154. doi: 10.1186/s12936-023-04574-7 (PMC10182553; doi:10.1186/s12936-023-04574-7)
Supplement: Supplementary file 1 — Additional file 1. Table S1: Paired sample genotyping data. Tables S1a–S1h and Tables S2a–S2h and Figs. S1–S8 of results showing liver enzymes, serum bilirubin, urea, creatinine, glucose and glucose of children enrolled in the study. [file 12936_2023_4574_MOESM1_ESM.docx]

***Supplementary file 1***

**Table S1: Discrimination of Recrudescence and Re-Infection of PA & AL using *msp1*, *msp2* & *glurp* Genes**

| **S/N** | **Study ID** | **Day of**  **Recurrence** | **PD (day of 2^nd^ infection)** | **PCR** | **MSP-2** | | **MSP-1** | | | **GLURP (bp)** | **Remark** | **Remark** |
| --- | --- | --- | --- | --- | --- | --- | --- | --- | --- | --- | --- | --- |
|  |  |  |  | **3FAL** | **3D7 (bp)** | **FC27 (bp)** | **K1**  **(bp)** | **RO33**  **(bp)** | **MAD20**  **(bp)** |  | **3/3 Approach** | **2/3 Approach** |
|  | 003 AL | D0  D28 | 1,751/µL | +  + | 300  600 | 800  ----- | ---  --- | --  150 | 200  250 | 1,000  1,000 | Reinfection | Reinfection |
|  | 004 PA | D0  D28 | 962/µL | +  + | **400**  **400** | 800  400 | ---  200 | --  150 | --  -- | **1,000**  **1,000** | Reinfection | Recrudescence |
|  | 035 AL | D0  D28 | 115/µL | +  + | 400, **800**  **800** | 700, 200  ------ | 200  --- | --  -- | --  200 | **1,000**  **1,000** | Reinfection | Recrudescence |
|  | 036 PA | D0  D21 | 17,676/µL | +  + | -------  400 | 400  500 | ---  200 | --  -- | 200  200 | 800, 900  1,000 | Reinfection | Reinfection |
|  | 041 AL | D0  D16 | 30,174/µL | +  + | 400, 700  ------ | -------  ------- | --  --- | --  150 | --  -- | 1,000  ------- | Reinfection | Reinfection |
|  | 042 AL | D0  D28 | 80/µL | +  + | **300,** 800  **300** | 400, 800  ------- | ---  200 | **150**  **150** | --  200 | 800  900 | **Reinfection** | **Recrudescence** |
|  | 043 PA | D0  D28 | 235/µL | +  + | 300  300 | 400  ------- | 150  200 | 150  -- | 200  -- | 900  700 | Reinfection | Reinfection |
|  | 052 PA | D0  D28 | 473/µL | +  + | 300  300 | -------  ------- | 200  250 | --  150 | --  250 | -------  800 | Reinfection | Reinfection |
|  | 055 AL | D0  D21 | 194/µL | +  + | 500  400 | -------  500 | 200  200 | --  -- | 300  200, 300 | 1,000  ------ | Reinfection | Reinfection |
|  | 057 AL | D0  D28 | 8,710/µL | +  + | **300**  **300** | 400  ------ | --  200 | **150**  **150** | 200  -- | 800  800 | **Recrudescence** | **Recrudescence** |
|  | 067  AL | D0  D28 | 158/µL | +  --- | 300, 500  -------- | 400  ------- | --  200 | 150  -- | 100, 200  -- | 1,000  ------ | Reinfection | Reinfection |
|  | 079 AL | D0  D21 | 88,700/µL | +  + | 300  **300,** 800 | 300  500 | ---  --- | --  -- | --  200 | 1,000  1,000 | Reinfection | Recrudescence |
|  | 080 AL | D0  D21 | 2,761/µL; | +  + | 500, 800  800 | 300, 400, 600  400 | ---  --- | --  150 | 200  -- | 700, 800  1,000 | Reinfection | Reinfection |
|  | 084 AL | D0  D28 | 2,640/µL | +  + | 300  400 | --------  ------- | 200  --- | 150  150 | --  200 | 800  700 | Reinfection | Reinfection |
|  | 086 PA | D0  D21 | 440/µL | +  + | 600  500 | **400**  **400** | 200  250 | --  -- | --  100, 200 | **1,000**  900, **1,000** | Recrudescence | **Recrudescence** |
|  | 090 AL | D0  D21 | 42,400/µL | +  + | 300  ------ | -------  ------- | --  200 | --  -- | 100, 200  100, 200 | 1,000  900 | Reinfection | Reinfection |
|  | 091 AL | D0  D 21 | 237/µL | +  + | 200  400 | 300  500 | 200  200 | 150  150 | 200  100, 200 | 800  900 | Reinfection | Reinfection |
|  | 094 AL | D0  D28 | 8436/µL | +  + | 300  500 | 400  ------- | 200  200 | 150  -- | **100, 200**  **100, 200** | 1,000  900 | Reinfection | Reinfection |
|  | 095 AL | D0  D21 | 1,778/µL | +  + | **300,** 400  **300** | 200,400, 500  200. 500 | **200**  **200** | --  -- | --  -- | **1,000**  **1,000** | Recrudescence | Recrudescence |
|  | 105 AL | D0  D28 | 33,261/µL | +  + | 400, 800  -------- | **500**  **500** | --  -- | --  -- | **100, 200**  **100, 200** | 900, **1000**  **1,000** | Recrudescence | Recrudescence |
|  | 134 AL | D0  D21 | 1,477/µL | +  + | **400,** 500  **400** | ----  ---- | --  200 | --  -- | 100, 200  -- | **900**  **900** | Reinfection | Recrudescence |
|  | 136  AL | D0  D28 | 452/µL | +  + | 300  400 | 500  ------- | 250  200 | --  150 | 100, 200  -- | 700  800 | Reinfection | Reinfection |
|  | 149 AL | D0  D21 |  | +  + | -----  200, 400 | 400, 800  ------- | ----  200 | 150  100 | --  -- | 1,000  800 | Reinfection | Reinfection |
|  | 158 AL | D0  D28 | 253/µL | +  + | ----  500 | **400**, 800  **400** | --  200 | --  -- | --  -- | **1,000**  **1,000** | Reinfection | Recrudescence |
|  | 167 PA | D0  D28 | 23,105/µL | +  + | **300,** 700  **300** | 300, 600  -------- | 200  --- | --  -- | 200  -- | 800  ------ | Reinfection | Reinfection |
|  | 171 AL | D0  D28 | 77077 | + | 300, 700  400 | 400  300 | --  200 | --  150 | 100, 200  100,200 | 1000  900 | Reinfection | Reinfection |
|  | 172 AL | D0  D28 | 160 | + | 400  300 | **400**  **400** | --  -- | --  -- | 100, 200  -- | 1000  ----- | Reinfection | Reinfection |
|  | 175 AL | D0  D26 | 118400 | + | 300  300 | 300  300 | 250  200 | 200  150 | --  -- | 1000  800 | Reinfection | Reinfection |
|  | 180 AL | D0  D28 | 17512 | + | ------  300 | 400  300 | 200  250 | 200  150 | 200  -- | 700  800 | Reinfection | Reinfection |
|  | 184 AL | D0  D28 | 90794 | + | 300  300 | -----  ----- | --  -- | --  150 | --  -- | 900  700 | Reinfection | Reinfection |

**AL = Artemether-Lumefantrine; PA = Pyronaridine Artesunate**

**Table S1a: Serum Urea pattern among children suffering from acute uncomplicated malaria treated with pyronaridine-artesunate or artemether-lumefantrine in Ibadan SW Nigeria**

| Characteristics on study days | Serum urea level (mg/dl) in drug groups | | | ρ-value |
| --- | --- | --- | --- | --- |
|  | PA | AL | Total |  |
| *Day 0*   - Mean (±sd) - Range | 20.75±9.07  10 – 55 | 23.52±10.45  10 – 68 | 22.12±9.83  10 – 68 | 0.126 |
| - No with raised value | 3 | 3 | 6 | 1.000 |
| - *No with ≥ 2ce ULN* | *NIL* | *NIL* | *NIL* | *---* |
| *Day 3*   - Mean (±sd) - Range | 18.76±7.82  9 – 50 | 18.64±7.47  10 – 39 | 18.70±7.62  9 – 50 | 0.698 |
| - No with raised value | 1 | 0 | 1 | 1.000 |
| - *No with ≥ 2ce ULN* | *NIL* | *NIL* | *NIL* | *---* |
| *Day 7*   - Mean (±sd) - Range | 18.62±7.24  10 – 42 | 18.41±6.99  10 – 41 | 18.51±7.09  10 – 42 | 0.859 |
| - No with raised value | NIL | NIL | NIL | -- |
| - *No with ≥ 2ce ULN* | *NIL* | *NIL* | *NIL* | *---* |
| *Day 28*   - Mean (±sd) - Range | 19.21±7.20  8 - 57 | 18.53±7.6  9 - 52 | 16.87±7.38  8 - 57 | 0.527 |
| - No with raised value | 1 | 2 | 3 | 0.613 |
| - *No with ≥ 2ce ULN* | *NIL* | *NIL* | *NIL* | *---* |

*Normal laboratory range of serum Urea 15-45mg/dl Normal value*

Bonferroni correction was made by setting the level of significance threshold at α divided by the number of hypothesis tests (4); p = 0.013

**Table S1b: Serum Creatinine pattern among children suffering from acute uncomplicated malaria treated with pyronaridine-artesunate or artemether-lumefantrine in Ibadan SW Nigeria**

| Characteristics on study days | Serum creatinine level in mg/dl in drug groups | | | ρ-value |
| --- | --- | --- | --- | --- |
|  | PA | AL | Total |  |
| *Day 0*   - Mean (±sd) - Range | 0.72±0.29  0.1 – 2.0 | 0.79±0.30  3 – 2.0 | 0.754±0.30  1 – 2.0 | 0.107 |
| No with raised value | 1 | 1 | 2 | 0.691 |
| *No with ≥ 2ce ULN* | *NIL* | *NIL* | *NIL* | *NIL* |
| *Day 3*   - Mean (±sd) - Range | 0.64±0.21  0.3 – 1.8 | 0.65±0.23  0.3 – 1.5 | 0.64±0.22  0.2 – 1.8 | 0.985 |
| - No with raised value | 1 | 0 | 1 | 0.547 |
| - *No with ≥ 2ce ULN* | *NIL* | *NIL* | *NIL* | *---* |
| *Day 7*   - Mean (±sd) - Range | 0.64±0.21  0.3 – 1.3 | 0.64±0.23  0.3 – 1.5 | 0.64±0.22  0.3 – 1.5 | 0.886 |
| - No with raised value | NIL | NIL | NIL |  |
| - *No with ≥ 2ce ULN* | *NIL* | *NIL* | *NIL* | *---* |
| *Day 28*   - Mean (±sd) - Range | 0.68±0.26  0.2 – 1.8 | 0.63±0.21  0.2 – 1.3 | 0.66±0.23  0.2 – 1.8 | 0.144 |
| - No with raised value | 1 | 0 | 1 | 0.368 |
| - *No with ≥ 2ce ULN* | *NIL* | *NIL* | *NIL* | *---* |

*Normal laboratory range of serum Creatinine level = 0.5 – 1.5 mg/dl*

**Table S1c: Serum Random Blood Glucose pattern among children suffering from acute uncomplicated malaria treated with pyronaridine-artesunate or artemether-lumefantrine in Ibadan SW Nigeria**

| Characteristics on study days | Serum blood sugar in mg/dl | | | ρ-value |
| --- | --- | --- | --- | --- |
|  | PA | AL | Total |  |
| *Day 0*   - Mean (±sd) - Range | 98.95±22.16  49 – 158 | 96.68±24.54  33 – 160 | 97.8±23.35  33 – 160 | 0.197 |
| - No with raised random serum blood sugar level | 4 | 5 | 9 | 0.342 |
| - *No with ≥ 2ce ULN* | *NIL* | *NIL* | *NIL* | *---* |
| *Day 3*   - Mean (±sd) - Range | 98.33±18.4  36 – 143 | 97.3±23.6  38 – 157 | 97.32±21.1  36 – 157 | 0.986 |
| - No with raised random serum blood sugar level | 0 | 2 | 2 | 0.217 |
| - *No with ≥ 2ce ULN* | *NIL* | *NIL* | *NIL* | *---* |
| *Day 7*   - Mean (±sd) - Range | 96.08±18.4  51 – 150 | 97.81±23.09  53 – 165 | 96.96±20.86  51 – 165 | 0.590 |
| - No with raised random serum blood sugar level | 3 | 6 | 9 | 0.623 |
| - *No with ≥ 2ce ULN* | *NIL* | *NIL* | *NIL* | *---* |
| *Day 28*   - Mean (±sd) - Range | 93.07±19.31  61- 144 | 95.70±19.51  56 – 159 | 94.39±19.39  56 – 159 | 0.394 |
| - No with raised random serum blood sugar level | 1 | 1 | 2 | 0.605 |
| - *No with ≥ 2ce ULN* | *NIL* | *NIL* | *NIL* | *---* |

Normal laboratory range of plasma random blood sugar = Normal – 60 – 140mg/dl

Bonferroni correction was made by setting the level of significance threshold at α divided by the number of hypothesis tests (4); p = 0.013

**Table S1d: Serum Total Bilirubin pattern among children suffering from acute uncomplicated malaria treated with pyronaridine-artesunate or artemether-lumefantrine in Ibadan SW Nigeria**

| Characteristics on study days | Total Serum Bilirubin in mg/dl | | | ρ-value |
| --- | --- | --- | --- | --- |
|  | PA | AL | Total |  |
| *Day 0*   - Mean (±sd) - Range | 0.63 ± 0.27  0.5 – 2.0 | 0.63 ± 0.26  0.3 – 1.9 | 0.63±0.26  0.3 – 20.0 | 0.969 |
| - No with raised total bilirubin level | 1 | 3 | 4 | 0.369 |
| - *No with ≥ 2ce ULN* | *1* | *0* | *1* | *---* |
| *Day 3*   - Mean (±sd) - Range | 0.62 ± 0.12  0.3 – 1.1 | 0.60 ± 0.17  0.4 – 1.3 | 0.61 ± 0.18  0.3 – 1.3 | 0.534 |
| - No with raised total bilirubin level | 1 | 2 | 3 | 1.000 |
| - *No with ≥ 2ce ULN* | *NIL* | *NIL* | *NIL* | *---* |
| *Day 7*   - Mean (±sd) - Range | 0.60±0.21  0.3 – 1.3 | 0.58±0.19  2 – 1.4 | 0.59±0.20  0.2 – 1.4 | 0.511 |
| - No with raised total bilirubin level | 1 | 2 | 3 | 1.000 |
| - *No with ≥ 2ce ULN* | *NIL* | *NIL* | *NIL* | *---* |
| *Day 28*   - Mean (±sd) - Range | 0.57±0.17  0.2 - 1.1 | 0.60±0.18  0.3 – 1.1 | 0.59±0.18  0.2 – 1.1 | 0.280 |
| - No with raised total bilirubin level | 1 | 3 | 4 | 0.367 |
| - *No with ≥ 2ce ULN* | *0* | *1* | *1* | *---* |

*Normal laboratory range of total serum bilirubin = 0.2 – 1.0mg/dl*

Bonferroni correction was made by setting the level of significance threshold at α divided by the number of hypothesis tests (4); p = 0.013

**Table S1e: Serum Conjugated Bilirubin pattern among children suffering from acute uncomplicated malaria treated with pyronaridine-artesunate or artemether-lumefantrine in Ibadan SW Nigeria**

| Characteristics on study days | Serum conjugated bilirubin level in mg/dl | | | ρ-value |
| --- | --- | --- | --- | --- |
|  | PA | AL | Total |  |
| D0   - Mean (±sd) - Range | 0.31±0.11  0.1 – 0.5 | 0.31±0.12  0.1 – 0.9 | 0.31±0.12  0.1 – 0.9 | 0.866 |
| - No with raised conjugated bilirubin level | 7 | 9 | 16 | 0.794 |
| - *No with ≥ 2ce ULN* | *0* | *1* | *1* | *---* |
| *Day 3*   - Mean (±sd) - Range | 0.30±0.10  0.1 – 0.6 | 0.29±0.11  0.1 – 0.8 | 0.30±0.11  0.1 – 0.8 | 0.532 |
| - No with raised conjugated bilirubin level | 6 | 5 | 11 | 1.000 |
| - *No with ≥ 2ce ULN* | *0* | *1* | *1* | *---* |
| *Day 7*   - Mean (±sd) - Range | 0.3±0.12  0.1 – 0.6 | 0.3±0.14  0.1 – 0.8 | 0.3±0.13  0.1 – 0.8 | 0.948 |
| - No with raised conjugated bilirubin level | 12 | 10 | 22 | 0.820 |
| - *No with ≥ 2ce ULN* | 0 | 1 | 1 | -- |
| *Day 28*   - Mean (±sd) - Range | 0.30±0.11  0.1 - 0.7 | 0.28±0.12  0.1 – 0.6 | 0.28±0.12  0.1 – 0.7 | 0.870 |
| - No with raised conjugated bilirubin level | 5 | 9 | 14 | 0.278 |
| - *No with ≥ 2ce ULN* | *NIL* | *NIL* | *NIL* | *NIL* |

*Normal laboratory range of serum conjugated bilirubin = 0 – 0.4/dl*

Bonferroni correction was made by setting the level of significance threshold at α divided by the number of hypothesis tests (4); p = 0.013

**Table S1f: Aspartate transaminase (AST) pattern among children suffering from acute uncomplicated malaria treated with pyronaridine-artesunate or artemether-lumefantrine in Ibadan SW Nigeria**

| Characteristics on study days | Serum level of aspartate Transaminases in IU/L | | | ρ-value |
| --- | --- | --- | --- | --- |
|  | PA | AL | Total |  |
| *Day 0*   - Mean ±sd - Range | 22.21±8.04  7 – 44 | 20.98±11.58  5 – 92 | 21.59±10.00  5 – 92 | 0.419 |
| - *No with raised serum AST* | *2* | *3* | *5* | *1.000* |
| - *AST ≥ 2ce ULN* | *0* | *1* | *1* | *--* |
| *Day 3*   - Mean±sd - Range | 22.39±8.55  7 – 75 | 20.94±11,96  7 – 46 | 21.53±10.02  7 – 75 | 0.208 |
| - *No with raised serum AST* | *4* | *3* | *7* | *0.720* |
| - *• AST ≥ 2ce ULN* | *1* | *0* | *1* | *--* |
| *Day 7*   - Mean±sd - Range | 21.36±8.90  2 – 58 | 20.94±11.06  3 – 76 | 20.15±10.02  2 – 76 | 0.783 |
| - *No with raised serum AST* | *3* | *4* | *7* | *1.000* |
| - *AST ≥ 2ce ULN* | *0* | *1* | *1* | *--* |
| *Day 28*   - Mean±sd - Range | 22.46±10.27  4 – 66 | 18.95±6.59  5 – 43 | 20.70±8.78  4 – 66 | 0.011 |
| - *No with raised Serum AST* | *5* | *1* | *6* | *0.211* |
| - *AST ≥ 2ce ULN* | *NIL* | *NIL* | *NIL* | *--* |

*Normal laboratory range of serum AST = 0 – 37 in IU/L*

Bonferroni correction was made by setting the level of significance threshold at α divided by the number of hypothesis tests (4); p = 0.013.

**Table S1g: Alanine transaminase (ALT) pattern among children suffering from acute uncomplicated malaria treated with pyronaridine-artesunate or artemether-lumefantrine in Ibadan SW Nigeria**

| Characteristics on study days | Serum ALT in IU/L | | | ρ-value |
| --- | --- | --- | --- | --- |
|  | PA | AL | Total |  |
| *Day 0*   - Mean (±sd) - Range | 19.95±7.65  4 – 40 | 20.55±14.18  3 – 97 | 20.25±11.38  3 – 97 | 0.734 |
| - *No with raised ALT* | *0* | *4* | *4* | *0.121* |
| - *ALT ≥ 2ce ULN* | *NIL* | *2* | *2* | *NA* |
| *Day 3*   - Mean (±sd) - Range | 20.06±9.07  3 – 49 | 18.82 ±7.81  5 – 52 | 19.42±8.90  3 – 52 | 0.361 |
| - *No with raised ALT* | *3* | *1* | *4* | *0.621* |
| - *ALT ≥ 2ce ULN* | *NIL* | *NIL* | *NIL* | *NIL* |
| *Day 7*   - Mean (±sd) - Range | 19.71±8.44  4 – 45 | 18.77±9.35  5 – 48 | 19.23+ 8.90  4 – 48 | 0.492 |
| - *No with raised ALT* | *1* | *4* | *5* | *0.019* |
| - *ALT ≥ 2ce ULN* | *NIL* | *NIL* | *NIL* | *NIL* |
| *Day 28*   - Mean (±sd) - Range | 20.00±8.92  4 – 54 | 17.21±5.67  5 – 34 | 18.60±7.58  4 – 54 | 0.019 |
| - *No with raised ALT* | *1* | *0* | *1* | *1.000* |
| - *ALT ≥ 2ce ULN* | *NIL* | *IL* | *NIL* | *NIL* |

*Normal laboratory range of serum ALT = 0 – 40 in IU/L*

Bonferroni correction was made by setting the level of significance threshold at α divided by the number of hypothesis tests (4); p = 0.013

**Table S1h: Alkaline phosphatase (ALP) pattern among children suffering from acute uncomplicated malaria treated with pyronaridine-artesunate or artemether-lumefantrine in Ibadan SW Nigeria**

| Characteristics on study days | Serum ALP in IU/L | | | ρ-value |
| --- | --- | --- | --- | --- |
|  | PA | AL | Total |  |
| *Day 0*  Mean ± sd  Range | 68.85±10.85  48 – 101 | 67.03±14.61  30 – 125 | 67.69±12.80 30 – 125 | 0.504 |
| *No with raised ALP* | *0* | *2* | *2* | *0.497* |
| *• ALP ≥ 2ce ULN* | *NIL* | *NIL* | *NIL* | *NIL* |
| *Day 3*   - Mean ± sd - Range | 68.07±13.21  35 – 109 | 67.73±13.94  25 –128 | 67.90±13.54  25 –128 | 0.871 |
| - *No with raised ALP* | *2* | *2* | *4* | *0.601* |
| - *ALP ≥ 2ce ULN* |  | *NIL* | *NIL* | *NIL* |
| *Day 7*  Mean ± sd  Range | 68.01±12.21  43 – 100 | 67.60±13.14  44 – 110 | 67.81+ 12.65  43 – 110 | 0.984 |
| *No with raised ALP* | *0* | *2* | *2* | *0.497* |
| *• ALP ≥ 2ce ULN* | *NIL* | *NIL* | *NIL* | *NIL* |
| *Day 28*  Mean ± sd  Range | 71.36±13.35  50 – 116 | 66.20±8.26  54 – 99 | 68.76±11.35  50 – 119 | 0.003 |
| - *No with raised ALP* | *1* | *0* | *1* | *0.497* |
| - *• ALP ≥ 2ce ULN* | *NIL* | *NIL* | *NIL* | *NIL* |

*Normal laboratory range for serum ALP = 35 – 105 in IU/L*

Bonferroni correction was made by setting the level of significance threshold at α divided by the number of hypothesis tests (4); p = 0.013

**Table S2a: Post-Hoc Comparisons of Mean Serum Urea Between PA And AL By Days of Follow Up**

| Days of follow up | Mean Difference | SE | t | ρ |
| --- | --- | --- | --- | --- |
| Days 0 vs 3 | 3.461 | 0.659 | 5.256 | < 0.001 |
| Days 0 vs 7 | 3.669 | 0.725 | 5.057 | < 0.001 |
| Days 0 vs 28 | 3.056 | 0.889 | 3.438 | 0.005 |
| Days 3 vs 7 | 0.208 | 0.534 | 0.389 | 1.000 |
| Days 3 vs 28 | -0.405 | 0.757 | -0.535 | 1.000 |
| Days 7 vs 28 | -0.613 | 0.619 | -0.991 | 1.000 |

Bonferroni correction was made by setting the level of significance threshold at α divided by the number of hypothesis tests (4); p = 0.013


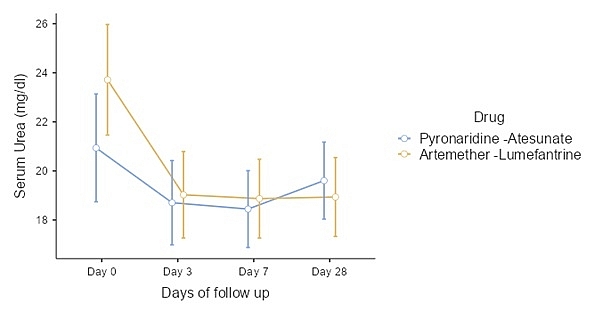


**Figure S1: Mean *of* Serum Urea of *Participants in the PA and AL Groups by days of follow up***

**Table S2b: Post Hoc Comparisons of Serum Creatinine Mean Difference Between PA and AL by Days of Follow Up**

| Days of follow up | Mean Difference | SE | t | ρ |
| --- | --- | --- | --- | --- |
| Days 0 vs 3 | 0.11665 | 0.0233 | 0.0233 | < 0.001 |
| Days 0 vs 7 | 0.10967 | 0.0259 | 0.0259 | < 0.001 |
| Days 0 vs 28 | 0.10420 | 0.0277 | 0.0277 | 0.001 |
| Days 3 vs 7 | -0.00698 | 0.0210 | 0.0210 | 1.000 |
| Days 3 vs 28 | -0.01244 | 0.0263 | 0.0263 | 1.000 |
| Days 7 vs 28 | -0.00547 | 0.0215 | 0.0215 | 1.000 |

Bonferroni correction was made by setting the level of significance threshold at α divided by the number of hypothesis tests (4); p = 0.013


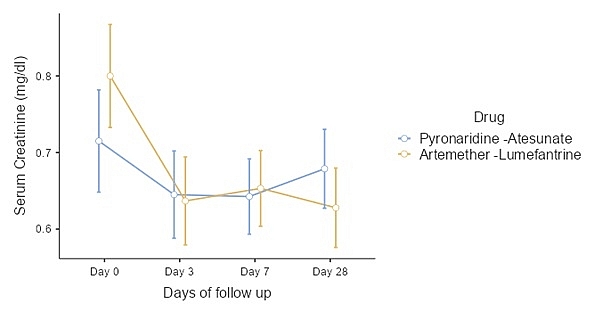


**Figure S2: Mean *of* Serum Creatinine of *Participants in the PA and AL Groups by days of follow up***

**Table S2c: Post Hoc Comparisons of Blood Glucose Mean Difference Between PA and AL by Days of Follow Up**

| Days of follow up | Mean Difference | SE | t | ρ |
| --- | --- | --- | --- | --- |
| Days 0 vs 3 | 0.02498 | 0.0187 | 1.333 | 1.000 |
| Days 0 vs 7 | 0.04484 | 0.0209 | 2.149 | 0.199 |
| Days 0 vs 28 | 0.04038 | 0.0201 | 2.004 | 0.280 |
| Days 3 vs 7 | 0.01985 | 0.0183 | 1.087 | 1.000 |
| Days 3 vs 28 | 0.01539 | 0.0154 | 0.999 | 1.000 |
| Days 7 vs 28 | -0.00446 | 0.0172 | -0.259 | 1.000 |

Bonferroni correction was made by setting the level of significance threshold at α divided by the number of hypothesis tests (4); p = 0.013


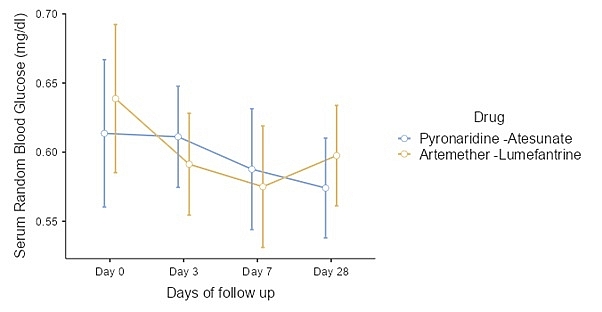


**Figure S3: Mean *of* Blood Glucose of *Participants in the PA and AL Groups by days of follow up***

**Table S2d: Post Hoc Comparisons of Serum Total Bilirubin Mean Difference Between PA and AL by Days of Follow Up**

| Days of follow up | Mean Difference | SE | t | ρ |
| --- | --- | --- | --- | --- |
| Days 0 vs 3 | 0.02498 | 0.0187 | 1.333 | 1.000 |
| Days 0 vs 7 | 0.04484 | 0.0209 | 2.149 | 0.199 |
| Days 0 vs 28 | 0.04038 | 0.0201 | 2.004 | 0.280 |
| Days 3 vs 7 | 0.01985 | 0.0183 | 1.087 | 1.000 |
| Days 3 vs 28 | 0.01539 | 0.0154 | 0.999 | 1.000 |
| Days 7 vs 28 | -0.00446 | 0.0172 | -0.259 | 1.000 |

Bonferroni correction was made by setting the level of significance threshold at α divided by the number of hypothesis tests (4); p = 0.013


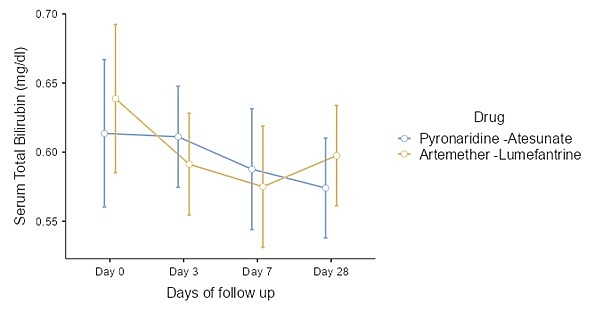


**Figure S4: Mean *of* Serum Total Bilirubin of *Participants in the PA and AL Groups by days of follow up***

**Table S2e: Post Hoc** **Comparisons of Serum Conjugated Bilirubin Mean Difference Between PA and AL by Days of Follow Up**

| Days of follow up | Mean Difference | SE | t | ρ |
| --- | --- | --- | --- | --- |
| Days 0 vs 3 | 0.01258 | 0.00894 | 1.407 | 0.968 |
| Days 0 vs 7 | 0.01624 | 0.01080 | 1.503 | 0.809 |
| Days 0 vs 28 | 0.02073 | 0.01048 | 1.978 | 0.298 |
| Days 3 vs 7 | 0.00366 | 0.01031 | 0.355 | 1.000 |
| Days 3 vs 28 | 0.00814 | 0.01036 | 0.786 | 1.000 |
| Days 7 vs 28 | 0.00449 | 0.01075 | 0.417 | 1.000 |

Bonferroni correction was made by setting the level of significance threshold at α divided by the number of hypothesis tests (4); p = 0.013


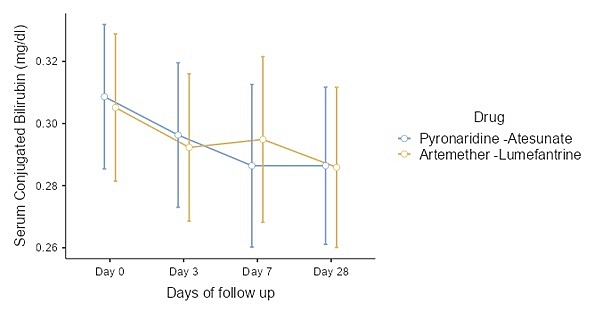


**Figure S5: Mean *of* Serum Conjugated Bilirubin of *Participants in the PA and AL Groups by days of follow up***

**Table S2f: Post Hoc Comparisons of Serum Aspartate Transaminase Mean Difference Between PA and AL by Days of Follow Up**

| Days of follow up | Mean Difference | SE | t | ρ |
| --- | --- | --- | --- | --- |
| Days 0 vs 3 | -0.00324 | 0.672 | -0.00483 | 1.000 |
| Days 0 vs 7 | 0.75309 | 0.853 | 0.88325 | 1.000 |
| Days 0 vs 28 | 0.86329 | 0.924 | 0.93399 | 1.000 |
| Days 3 vs 7 | 0.75633 | 0.854 | 0.88515 | 1.000 |
| Days 3 vs 28 | 0.86653 | 0.719 | 1.20482 | 1.000 |
| Days 7 vs 28 | 0.11021 | 0.855 | 0.12885 | 1.000 |

Bonferroni correction was made by setting the level of significance threshold at α divided by the number of hypothesis tests (4); p = 0.013


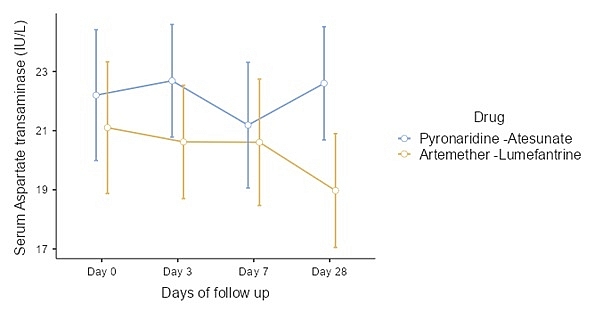


**Figure S6: Mean *of* Serum Aspartate Transaminase of *Participants in the PA and AL Groups by days of follow up***

**Table S2g: Post Hoc Comparisons of Serum Alanine Transaminase Mean Difference Between PA and AL by Days of Follow Up**

| Days of follow up | Mean Difference | SE | df | t | ρ |
| --- | --- | --- | --- | --- | --- |
| Days 0 vs 3 | 0.996 | 0.834 | 158 | 1.194 | 1.000 |
| Days 0 vs 7 | 1.208 | 0.862 | 158 | 1.402 | 0.977 |
| Days 0 vs 28 | 1.798 | 0.949 | 158 | 1.895 | 0.360 |
| Days 3 vs 7 | 0.212 | 0.692 | 158 | 0.306 | 1.000 |
| Days 3 vs 28 | 0.802 | 0.670 | 158 | 1.197 | 1.000 |
| Days 7 vs 28 | 0.590 | 0.704 | 158 | 0.838 | 1.000 |

Bonferroni correction was made by setting the level of significance threshold at α divided by the number of hypothesis tests (4); p = 0.013


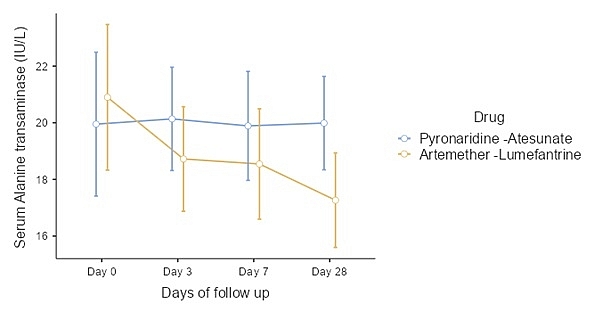


**Figure S7: Mean *of* Serum Alanine Transaminase of *Participants in the PA and AL Groups by days of follow up***

**Table S2h: Post-Hoc Comparisons of Serum Alkaline Phosphatase Mean Difference Between PA and AL by Days of Follow Up**

| Days of follow up | Mean Difference | SE | df | t | ρ |
| --- | --- | --- | --- | --- | --- |
| Days 0 vs 3 | -0.278 | 1.13 | 158 | -0.2454 | 1.000 |
| Days 0 vs 7 | -0.105 | 1.12 | 158 | -0.0940 | 1.000 |
| Days 0 vs 28 | -1.123 | 1.28 | 158 | -0.8773 | 1.000 |
| Days 3 vs 7 | 0.173 | 1.05 | 158 | 0.1646 | 1.000 |
| Days 3 vs 28 | -0.846 | 1.16 | 158 | -0.7274 | 1.000 |
| Days 7 vs 28 | -1.019 | 1.07 | 158 | -0.9501 | 1.000 |

 Bonferroni correction was made by setting the level of significance threshold at α divided by the number of hypothesis tests (4); p = 0.013


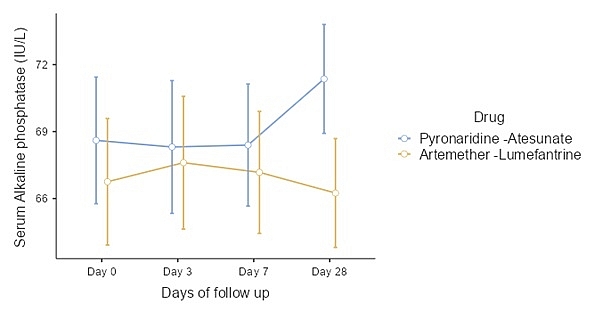


**Figure S8: Mean *of* Serum Alkaline Phosphatase *Participants in the PA and AL Groups by* Days of Follow Up**
