# Supplementary material for: Efficacy and safety of pyronaridine–artesunate versus artemether–lumefantrine in the treatment of acute uncomplicated malaria in children in South-West Nigeria: an open-labelled randomized controlled trial
Source: Malar J. 2023 May 13;22:154. doi: 10.1186/s12936-023-04574-7 (PMC10182553; doi:10.1186/s12936-023-04574-7)
Supplement: Supplementary file 2 — Additional file 2. Table S3: Tables S3a and S3b showing gametocyte carriage among children treated with artemether–lumefantrine and pyronaridine–artesunate during the study. [file 12936_2023_4574_MOESM2_ESM.docx]

**Supplementary file 2**

**Table S3a: Gametocyte carriage among Nigerian children suffering from acute uncomplicated malaria treated with Artemether-lumefantrine or Pyronaridine-artesunate with patent parasitaemia southwest Nigeria during the 28 days follow-up**

| Study Day | Drug Group | | | ρ-value |
| --- | --- | --- | --- | --- |
|  | AL [N (%)] | PA [N (%)] | Total  [N (%)] |  |
| Day 0 | 7 (8.0) | 14 (16 .5) | 21 (12.2) | 0.107 |
| Day 1 | 7 (8.0) | 14 (16.5) | 21 (12.2) | 0.108 |
| Day 2 | 6 (7.0) | 12 (12.9) | 18 (10.5) | 0.143 |
| Day 3 | 5 (5.9) | 8 (9.4) | 13 (7.6) | 0.586 |
| Day 7 | 0 (0.0) | 6 (7.1) | 6 (3.4) | 0.013 |
| Day 14 | 0 (0.0) | 4 (4.7) | 4 (2.5) | 0.120 |
| Day 21 | 0 (0.0) | 3 (3.7) | 3 (1.9) | 0.245 |
| Day 28 | 2 (2.5) | 1 (1.2) | 2 (1.2) | 0.618 |
| Gametocyte clearance time   - Mean ± sd (days) - Range (days) | 3.00 ± 1.16  1 – 4 | 8.43 ± 8.87  1 – 28 | 6.62 ±7.65  1 – 28 | 0.125 |
| - No with gametocyte present any day | 11 (12.6) | 17 (20.0) | 27 (15.7) | 0.219 |
| - No on D0 with gametocytes | 7 (8.0) | 14 (16.5) | 21 (12.2) | 0.107 |

**Table S3b: Characteristics of gametocyte carriage among children from southwest Nigeria suffering from acute uncomplicated malaria treated with Artemether-lumefantrine or Pyronaridine-artesunate during the 28 days follow-up**

| Gametocyte characteristics on  Study Day | Drug Group | | | ρ-value |
| --- | --- | --- | --- | --- |
|  | AL [N (%)] | PA [N (%)] | Total [N (%)] |  |
| *Day 0*   - No with gametocytes - Geomean (µL) - Range (µL) | 7  25.52  8-2400 | 14  33.52  8-2400 | 21  30.6  8 -2400 | 0.531 |
| *Day 1*   - No with gametocytes - Geomean (µL) - Range (µL) | 7  22.12  8-103 | 14  29.64  8.1880 | 21  26.88  8-1880 | 0.504 |
| *Day 2*   - No with gametocytes - Geomean (µL) - Range (µL) | 6  24.29  8 - 64 | 12  39.1  8 - 3088 | 18  33.36  8 - 3088 | 0.483 |
| *Day 3*   - No with gametocytes - Geomean (µL) - Range (µL) | 5  21.79  8 - 48 | 8  42.86  8 - 2096 | 13  33.04  8 - 2096 | 0.444 |
| *Day 7*   - No with gametocytes - Geomean (µL) - Range (µL) | 0  0  0 | 6  641  8 - 1600 | 6  641  8 - 1600 | NA |
| *Day 14*   - No with gametocytes - Geomean (µL) - Range (µL) | 0  0  0 | 4  55.1  8 - 1440 | 4  55.1  8 - 1440 | NA |
| *Day 21*   - No with gametocytes - Geomean (µL) - Range (µL) | 0  0  0 | 3  46.78  8 – 800 | 3  46.78  8 – 800 | NA |
| *Day 28*   - No with gametocytes - Geomean (µL) - Range (µL) | 2  32.0  8 - 128 | 1  728  728 | 3  90.67  8 - 728 | 0.099 |
